# *Drosophila* Genotype Influences Commensal Bacterial Levels

**DOI:** 10.1371/journal.pone.0170332

**Published:** 2017-01-17

**Authors:** Angela M. Early, Niroshan Shanmugarajah, Nicolas Buchon, Andrew G. Clark

**Affiliations:** 1 Department of Ecology and Evolutionary Biology, Cornell University, Ithaca, New York, United States of America; 2 Department of Molecular Biology and Genetics, Cornell University, Ithaca, New York, United States of America; 3 Department of Entomology, Cornell University, Ithaca, New York, United States of America; University of Illinois at Urbana-Champaign, UNITED STATES

## Abstract

Host genotype can influence the composition of the commensal bacterial community in some organisms. Composition, however, is only one parameter describing a microbial community. Here, we test whether a second parameter—abundance of bacteria—is a heritable trait by quantifying the presence of four commensal bacterial strains within 36 gnotobiotic inbred lines of *Drosophila melanogaster*. We find that *D*. *melanogaster* genotype exerts a significant effect on microbial levels within the fly. When introduced as monocultures into axenic flies, three of the four bacterial strains were reliably detected within the fly. The amounts of these different strains are strongly correlated, suggesting that the host regulates commensal bacteria through general, not bacteria-specific, means. While the correlation does not appear to be driven by simple variation in overall gut dimensions, a genetic association study suggests that variation in commensal bacterial load may largely be attributed to physical aspects of host cell growth and development.

## Introduction

Advances in microbiome research have demonstrated the need to consider the phenotypic effects of not only environmental conditions and organismal genotype, but also microbiome composition and by extension, the complex interactions among all three players. This holobiont concept has become an established paradigm in biology and has impacted diverse fields from physiology to evolution [1]. Studies have been conducted in a wide range of organisms and have uncovered relationships between commensal bacteria and a plethora of host traits from metabolism to behavior [2–5].

In the past decade, researchers have published nearly a dozen sequence-based surveys of *Drosophila*-associated microbes (reviewed in [6]). These studies have taken diverse approaches and examined the effects of food source, developmental stage, and various laboratory and natural environments. Contrary to initial expectations, however, these efforts uncovered no evidence of a well-defined core microbiome at the species level [7, 8]. Instead, the composition of the fly microbiome is strongly affected by environmental factors such as food substrate [7, 9], and its maintenance is likely dependent on constant replenishment through the ingestion of environmental microbes [10]. Still, the fly’s microbiome is not a pure byproduct of the environment. Rather, it is becoming increasingly clear that *Drosophila* exerts a certain degree of selective regulation—both directly and indirectly—over its microbiome composition. Only a small subset of the microbes encountered by the fly survive within the gut [9], and there are certain bacterial taxa that are repeatedly sampled across *Drosophila* species and habitats. These include the genera *Acetobacter*, *Lactobacillus*, *Gluconobacter*, and *Enterococcus*, which are all acid-tolerant bacteria that can survive in the gut’s low pH [7, 9, 11–13]. Recent evidence further shows that fly genotype affects the relative abundance of observed bacterial strains [14], leading to natural variation in the composition of fly gut bacterial communities within the same environment.

While *Drosophila* has no obligate gut microbe, the presence of a microbiome does have fitness consequences for the host. When gut microbes are experimentally removed in the laboratory, the resulting axenic flies are viable and experience various fitness effects including metabolic dysregulation [15], decreased intestinal aging [16], altered lifespan [16, 17], and enhanced susceptibility to oral pathogens [10, 18]. In addition, specific bacterial strains have been associated with a variety of processes including insulin signaling [15], growth and development [19, 20], and even mating preference [21]. The apparently loose relationship between *Drosophila* and specific microbes therefore raises an intriguing question that is relevant to a broad array of taxa [22]. In the absence of strong co-evolutionary relationships, how do hosts optimize the benefits they derive—or at least minimize the harm they receive—from transient microbial partners?

One important answer to this question is likely host regulation of bacterial growth. Indeed, perturbing flies’ natural regulation of gut bacteria in either direction is harmful [23–26]. Several aspects of the fly’s gut physiology and immune response are known to play roles in this microbial regulation [27]. First, a low pH and the presence of digestive enzymes create an environment that is inhospitable to many bacteria [28, 29]. Second, the peritrophic matrix, a chitinous lining in the midgut, serves as a physical barrier, blocking microbial access to the epithelium [30]. Third, a gut-specific immune response places a check on microbial proliferation through the release of reactive oxygen species (ROS) [25] and antimicrobial peptides (AMPs) [31]. While we are forming a more comprehensive picture of how these processes respond to pathogenic infection [32], we still know little about how the gut regulates commensal bacterial communities and maintains homeostasis [19].

We propose that one key—and hitherto uninvestigated—aspect of the fly-microbiome relationship is the relative size of the microbial community. Here we test whether fly genotype influences not the composition, but the size of the internal microbial population. We find that this trait does vary among flies in a heritable fashion and is largely robust to different bacterial genotypes.

## Results

### Drosophila haplotypes harbor commensal bacteria populations of variable size

To test whether fly genotype affects the quantity of retained commensal bacteria, we created sets of gnotobiotic fly lines that were each colonized by a single bacterial strain. In total, we used 36 fly lines from the Drosophila Genetic Reference Panel (DGRP) [33], a set of inbred fly lines sampled from a single population in North Carolina. For each fly genotype, we created sets of four gnotobiotic lines that were colonized with a single bacterial strain that is known to reside in the fly gut (*Acetobacter tropicalis*, *Enterococcus faecalis*, *Lactobacillus brevis*, or *L*. *plantarum*). Three of these bacterial strains (*A*. *tropicalis*, *L*. *brevis*, and *L*. *plantarum*) were directly isolated from laboratory fly stocks and were previously shown to be dominant members of the microbial gut community in laboratory flies [13]. After inoculating an axenic parental generation with the focal bacterium, we reared offspring in an environment where they were exposed to only this single bacterial strain from the egg to adult stage. We then measured bacterial levels in 3–5 day old adult males from this generation of flies using quantitative PCR. Under natural conditions, the fly gut would harbor a bacterial community, not a single strain. Within such a community, individual strain abundance would be determined by a combination of three main factors: environment, host genotype, and microbial community composition. By maintaining a controlled environment and eliminating competition among bacterial strains, we were here able to measure the host effect in isolation.

**Fig 1:**
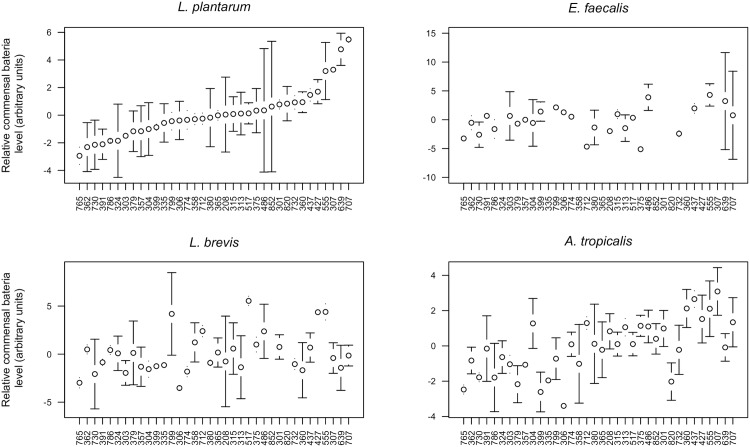
Relative size of commensal bacteria populations in 36 inbred fly lines. Relative commensal bacterial level was determined using quantitative PCR and was calculated relative to levels of a single copy *Drosophila* gene (Drosophila gene Ct—Bacterial gene Ct). The y-axis shows the residuals of these measurements from a model accounting for block effect (mean ± 1 S.D.). Units are on a log2 scale. Higher values correspond to a higher ratio of bacterial DNA to fly DNA. In all plots, the lines are ordered according the rank order of the *L*. *plantarum* residuals.

For three of the four bacteria (*A*. *tropicalis*, *L*. *brevis*, and *L*. *plantarum*), we were able to measure significant bacterial population-size differences among fly lines (Fig 1; ANOVA, *A*. *tropicalis*, *P* = 0.0005, *L*. *brevis*, *P* = 0.0008; *L*. *plantarum*, *P* = 0.0007). No significant line effect was detected for the fourth bacterial strain, *E*. *faecalis* (ANOVA, *P* = 0.462). This bacterium was detected in only a small subset of our samples (26 lines, 48 total samples), and so we likely lacked the power to make inter- and intra-line comparisons. To quantify the microenvironmental and genetic factors contributing to this inter-line variation, we calculated broad sense heritability (H^2^), which measures the extent to which a phenotype is attributable to genetic versus environmental causes. As in the ANOVA, H^2^ was substantial (>0.38) for *A*. *tropicalis*, *L*. *brevis*, and *L*. *plantarum*, but relatively low (0.091) for *E*. *faecalis* (Table 1). This shows that commensal bacteria level is influenced by fly genotype and is not a pure by-product of environmental conditions (Fig 1, S1 Table). The inability of *E*. *faecalis* to consistently establish associations with the flies is curious given that Cox and Gilmore [12] did create stable recolonizations with a different strain of *E*. *faecalis*. The discrepancy might reflect differences in the two strains. For instance, ours was isolated from the hemolymph, not the gut, of a wild-caught fly.

**Table 1:** Significant genetic variation and heritability in abundance of commensal gut bacteria across DGRP lines of *Drosophila*.

	d.f.	*F*	*P*	Genetic variance	Error variance	Block variance	H^2^
***A*. *tropicalis***	35	2.72	0.0005	1.46	2.24	0.0357	0.394
***E*. *faecalis***	25	1.03	0.462	0.853	8.24	2.45	0.0938
***L*. *brevis***	33	2.61	0.0008	2.74	4.40	0.000	0.384
***L*. *plantarum***	35	2.60	0.0007	1.92	3.04	12.8	0.388

Interestingly, we found that relative bacterial levels were significantly correlated in all but the comparisons with *E*. *faecalis* (Table 2). The three bacteria whose relative gut titer correlated are all major colonizers of *Drosophila*, however, there are known biological differences among them [6]. The strong correlation we see between their relative levels therefore suggests that the effect of fly genotype is consistent across disparate bacterial strains and not highly dependent on the composition of the microbial community. This observation is consistent with the idea of there being little species-specific bacterial regulation on the part of the fly.

**Table 2:** Correlations between relative commensal bacterial level.

	*A*. *tropicalis*	*E*. *faecalis*	*L*. *brevis*
***E*. *faecalis***	0.109		
***L*. *brevis***	0.411*	0.151	
***L*. *plantarum***	0.689***	0.335	0.666***

Spearman’s *ρ*.

*, *P < 0*.*05;*

**, P < 0.01;

***, *P* < 0.0001

Bacterial population size was calculated as the ratio of bacterial DNA to fly DNA as measured with qPCR. Correlations were performed on the residuals from a model that accounted for batch effect.

By culturing homogenates of single dissected guts colonized by *A*. *tropicalis*, we determined how the qPCR values (Fig 1) correspond to culture-dependent methods that measure colony forming units (CFUs). We found that mean per gut values for male flies ranged from 47 CFUs (line 306) to 3035 CFUs (line 307). As we transferred the flies to autoclaved media the morning before the measurements were made, these measurements are lower than those made with conventionally reared flies [10].

### Production of reactive oxygen species does not correlate with commensal bacterial levels

It is known that the gut’s production of reactive oxygen species (ROS) contributes to commensal bacteria regulation as perturbing flies’ basal production of ROS leads to dramatic changes in the growth of gut bacteria [25]. We reasoned that variation in ROS production may contribute to the variation we observed in commensal bacterial loads. Using a ferrous oxidation-xylenol orange (FOX) assay [34], we measured the oxidative capacity of freshly dissected guts from conventionally reared flies. We used male flies from 9 lines chosen from the two extremes of the *L*. *plantarum* load distribution. Levels of ROS varied significantly among lines (ANOVA, *P* < 0.001) but there was no correlation between ROS production and bacterial levels (S2 Table and S1 Fig).

### Results from association testing

Since ROS levels did not appear to be driving the variation in gut bacterial load, we performed a genetic association test to identify candidate genes that may underlie the phenotypic differences. Because of our small sample size, we did not expect to definitively identify the genetic architecture underlying this trait. We felt, however, that the analysis could suggest potential mechanisms worthy of future investigation. Using EMMAX [35], we tested for associations between 1,455,565 filtered variants and bacterial levels of *L*. *plantarum*, *L*. *brevis*, and *A*. *tropicalis*. At an FDR of 0.05, *L*. *plantarum* levels showed four genetic associations, *L*. *brevis* levels had 16 and *A*. *tropicalis* levels had none. After a more stringent Bonferroni correction for multiple testing, three SNPs remained statistically significant in the *L*. *plantarum* test at an α of 0.05. All three variants are in complete linkage disequilibrium. Of the three, one is a nonsynonymous variant in the gene *cdc14*. This phosphatase is a rate-limiting inhibitor of mitotic exit [36] and has been implicated in the control of cell proliferation in *Drosophila* larvae [37]. The remaining two significantly associated SNPs are an intron variant in *cdc14* and a synonymous coding variant in *Mur89F*, which encodes a putative mucin with a chitin-binding domain. Interestingly, both genes are positively regulated in the gut in response to the ingestion of *Pseudomonas entomophila*, suggesting that these genes are responsive to microbes [38]. No associations with *L*. *brevis* remained significant after Bonferroni correction, however, one nonsynonymous SNP was significant at an FDR of 0.05. This missense variant is in CG16854, a gene of unknown function that is strongly expressed in the enteroendocrine cells of the midgut and is also responsive to microbes [38]. A list of all variants significant at an FDR of 0.05 can be found in S3 Table.

### Gene enrichment analysis suggests that non-immune physiological factors govern variation in commensal bacteria levels

To further dissect the genetic architecture underlying this trait, we performed a Gene Ontology (GO) analysis using the most highly associated variants (*P* < 10^−5^). Using Ensembl annotations, we assigned each variant to a single gene. Combining the variants from the three association tests, we obtained a list of 99 genes. With Panther [39], we found that several GO categories are enriched in this gene set (Table 3). Interestingly, the categories showing significant fold enrichment are largely involved in neuronal function, neuronal morphogenesis and development, and general cellular growth and development. Of note, the gut epithelium comprises cells with neuronal identity, the enteroendocrine cells, that strongly influence gut physiology [40]. It is therefore possible that neuronal function reflects a role for the enteroendocrine lineage in the response to gut microbes. Results from DAVID [41] and GOrilla [42] led to similar interpretations (S4 Table).

**Table 3:** GO category enrichment of genes strongly associated with bacterial levels in the fly as calculated by Panther.

GO Biological Process, Experimental Only	Observed Gene Number	Expected Gene Number	Fold Enrichment	Bonferroni-corrected P-value
Movement of cell or subcellular component (GO:0006928)	16	3.43	4.66	5.97E-04
Axonogenesis (GO:0007409)	12	1.91	6.28	9.99E-04
Axon guidance (GO:0007411)	11	1.6	6.88	1.32E-03
Axon development (GO:0061564)	12	1.96	6.12	1.35E-03
Neuron projection guidance (GO:0097485)	11	1.65	6.67	1.77E-03
Cell morphogenesis involved in neuron differentiation (GO:0048667)	14	2.85	4.91	1.97E-03
Chemotaxis (GO:0006935)	11	1.7	6.47	2.44E-03
Anatomical structure morphogenesis (GO:0009653)	27	10.31	2.62	3.74E-03
Cell morphogenesis involved in differentiation (GO:0000904)	14	3.13	4.47	5.83E-03
Locomotion (GO:0040011)	15	3.65	4.11	7.03E-03
Neuron development (GO:0048666)	16	4.33	3.70	1.24E-02
Taxis (GO:0042330)	11	2.08	5.29	1.62E-02
Neuron differentiation (GO:0030182)	17	4.97	3.42	1.66E-02
Neuron projection morphogenesis (GO:0048812)	14	3.48	4.02	1.94E-02
Neuron projection development (GO:0031175)	14	3.57	3.92	2.58E-02
Generation of neurons (GO:0048699)	18	5.73	3.14	2.75E-02
Cell projection organization (GO:0030030)	15	4.2	3.57	3.72E-02
Tissue morphogenesis (GO:0048729)	15	4.24	3.54	4.10E-02
Cell projection morphogenesis (GO:0048858)	14	3.73	3.75	4.29E-02

* In total 99 significantly associated genes were included in the enrichment analysis

None of the enriched GO categories included immune processes, even though the epithelial immune response is known to play a role in regulating commensal bacteria growth [24]. As a final enrichment test, we therefore explicitly looked for genes implicated in the gut immune response. Since there is no GO category associated with this specific process, we assembled a list of 30 genes with known roles in the epithelial immune response. None of these genes were represented among our highly associated variants.

### Levels of commensal bacteria do not correlate with gut size

The results from the association study suggest that aspects of gut growth and development may drive inter-fly variation in gut bacterial levels. Our measurements of commensal bacterial titer were made relative to the number of cells in the fly’s body. Flies, however, might vary in the relative size of their guts, and we therefore explored whether variation in relative gut volume accounted for the observed variation in bacterial level. Under this model, flies with proportionally larger guts would be found supporting higher levels of bacteria simply because they carry a larger commensal habitat. For 28 conventionally reared fly lines, we obtained six measurements of midgut size: anterior width, posterior width, anterior length, middle length, posterior length, and total length. We found no correlation (*P* > 0.05) between any of these measurements and commensal bacterial levels. We note, however, that these gut measurements were from female flies whereas the bacterial levels were measured in male flies, and we cannot rule out sexual dimorphism in organ size. These concerns are partially alleviated by our previous observation that total midgut lengths in males and females do correlate (R^2^ = 0.8781, S2 Fig).

### Commensal bacterial titer correlates with an index of fly nutrient storage

Previous studies of these fly lines have measured a number of additional phenotypes including starvation resistance [43], recovery time from chill coma [43], life span [43], competitive fitness [43], oxidative stress [44], and nutritional indices [45]. To investigate the potential fitness effects of commensal bacterial level, we looked for correlations between our data and these relevant phenotypes.

Only one trait significantly correlated with *L*. *plantarum* titer: nutritional stores of flies when reared on a high glucose diet. Specifically, we found a correlation with *L*. *plantarum* titer and the second principal component (PC) calculated by Unckless, *et al*. [43] for a set of nutritional indices measured in these flies (ρ = -0.4988, *P* = 0.004756). This PC explained approximately 25% of the variance among their set of DGRP lines and had a loading of: -0.76 glucose content, 0.54 protein content, -0.3 glycogen content, 0.17 glycerol content, and 0.06 triglyceride content. A weaker, but still significant correlation existed between *L*. *plantarum* titer and glucose storage, the metric with the highest loading in this PC (Spearman’s rank correlation test; ρ = 0.3883, *P* = 0.03167).

### No evidence that high levels of commensal bacteria aid pathogenic bacterial resistance

The competitive inhibition of invading pathogens is one proposed benefit of maintaining a commensal microbial community [10]. Under this hypothesis, lines that maintain larger amounts of commensal bacteria would have a higher resistance against invading pathogens. To test this, we compared our results to a recent study that investigated these same lines’ variability in gut immunocompetence [32]. The data showed no evidence in support of the competitive inhibition hypothesis, as there was no correlation between levels of commensal bacteria and time to death after enteric infection with the pathogen *Pseudomonas entomophila*. Similarly, we observed no trend supporting the possible alternative hypotheses that (1) lines with higher immunocompetence have lower commensal load (perhaps due to high constitutive immune expression) or (2) flies with an intermediate commensal load have higher fitness during pathogenic attack. Of course, the relatively small sample size (n = 36) of our study constrains these statements to pertain to relatively large effects.

## Discussion

*Drosophila* has been proposed as an important model organism for studying gut physiology in general and host-microbe interactions in particular [19, 27]. To date, however, research efforts have focused solely on the composition or complete presence/absence of the microbial community. Here we highlight a third parameter—commensal bacterial level—and show that it is both genetically determined and variable in natural populations. To place it in context of other *Drosophila* phenotypes, the broad-sense heritability (H^2^) for commensal bacterial level is in the range of the H^2^ estimated for chill coma recovery (0.374) [43], alcohol sensitivity (0.42) [46] and food intake (0.45) [47]. In the three cases where bacteria were reliably detected in the fly, we found strong correlations between commensal bacterial levels (Table 2), suggesting that the effect of host genotype is relatively constant across bacterial taxa.

Interestingly, variation in the amount of bacteria present in the fly does not appear driven by variation in basal immune activity. Fly bacteria levels showed no correlation with ROS levels or gut immune resistance in conventionally reared flies [32]. Additionally, none of the 99 strongly associated genes have a known role in the epithelial immune response, although the most significant SNPs have been shown to be regulated in the gut in response to infection [38]. Previous work has demonstrated that the gut’s pathogen-triggered immune response is regulated separately from its basal immune activity [48]. The lack of correlation between commensal bacterial levels and pathogen resistance further supports the idea that these two processes are at least partially governed by different genetic mechanisms.

Instead of being driven by variation in immune function, variation in commensal bacterial level appears to be influenced by physical aspects of gut cell growth and development. The importance of gut epithelial renewal in the face of both pathogenic infection [32, 49] and commensal colonization [50] has been previously described. The need for epithelial renewal is driven by the production of oxidative compounds in the gut, and it is likely that flies vary not only in the amounts of ROS produced, but also in the ways they protect themselves from this oxidative damage. As the microbiota impact key aspects of gut morphology such as epithelial turnover, cell spacing and cell type [51], each hosts’ unique response may in turn reshape the microbial community. Our enrichment analysis hints that the feedback from host to microbe likely involves neuronal input in addition to physical structuring of the gut. Gut homeostasis relies on neuronal circuitry that regulates intestinal function and fly feeding behavior, as well as on the function of dedicated neuron-like epithelial cells: the enteroendocrine cells [52–54]. A connection between the fly’s enteric neurons, or enteroendocrine cells, and its microbiota has been posited previously but remains unexplored [38, 53].

Similarly, we still know little about how the absolute abundance of bacteria impacts host fitness. Clues, however, come from recent studies focused on the relative abundance of bacterial taxa. Even small changes in the relative abundance of bacterial strains can have a nutritional impact on the *Drosophila* host [55]. Further, the fly genotype influences the composition of the gut microbial community, mediating the overall nutritional effect [14]. It is therefore not unexpected that we found a correlation between levels of *L*. *plantarum* and fly glucose content. The correlation suggests that flies with higher gut bacteria levels also store higher amounts of glucose. This phenotype warrants further investigation to determine whether the link is causal and if so, the directionality of the relationship. Does the fly’s use of glucose modify the gut environment, enabling higher bacterial growth? Or conversely, does harboring a larger bacterial population change the nutrients available to the fly, altering its metabolism and glucose storage? Interestingly, we are not the first to find a tie between glucose and microbiota: Galenza et al. (2015) recently observed that dietary glucose levels impact the microbiota composition of flies. While this study only considered the presence/absence of bacteria, the potential role played by absolute bacterial level is also intriguing.

The naturally segregating variation that we observe suggests that, as with many complex traits, there is no single optimal strategy for regulating commensal bacterial levels. Previous work in *D*. *melanogaster* has shown that relative levels of certain bacterial strains correlate with healthy versus pathological gut states and that the immunological activity of the gut can push the fly from one condition to the other [24]. Our results further suggest that immune-independent mechanisms impact the state of the gut microbiota, influencing the overall size of the microbial community. Further investigation into the effects of the natural variation we describe here will greatly inform our understanding of host-microbiome relationships and the potential trade-offs inherent in maintaining resident microbial populations.

## Materials and Methods

### Fly lines and bacterial stocks

We chose 36 lines from the Drosophila Genetic Reference Panel (DGRP) [33], a set of inbred *D*. *melanogaster* lines sampled in Raleigh, NC, USA. To phenotype each fly line for the commensal bacterial titer within its gut, we created gnotobiotic lines that contained a single bacterial strain. Three of these strains (*Lactobacillus brevis*, *L*. *plantarum*, and *Acetobacter tropicalis*) were isolated from the guts of laboratory *Drosophila* stocks [13]. The fourth was a strain of *Enterococcus faecalis* that was isolated from the hemolymph of wild-caught flies [56]. Prior to the microbiota manipulations, we treated all the fly lines with tetracycline to clear them of the intracellular symbiont *Wolbachia pipientis*. *Wolbachia* has no known effect on gut microbiota, but the removal of this endosymbiont facilitated our detection of gut bacteria with qPCR. All fly lines were given the same tetracycline treatment regardless of their initial *Wolbachia* infection status. For seven generations, flies were maintained on standard glucose-yeast media to which we added 0.03% tetracycline. We then returned the flies to untreated media to which we added the carcasses of four dead untreated flies of the same genotype. This ensured that the vials were seeded with the flies’ original microbiota. Following restoration of the natural microbial environment, flies were maintained for at least four additional generations before being used in the gut colonization experiments. At the end of the treatment, we confirmed that *Wolbachia* had been cleared with a standard PCR targeting the *Wolbachia wsp* gene [57].

### Creation of gnotobiotic lines

To measure the bacterial titer within fly guts, we manipulated the bacterial content of our 36 DGRP lines. We raised a bacteria-free generation of each fly line by dechorionating eggs with bleach and transferring them to autoclaved media. After adult axenic flies emerged from these vials, we transferred them to 1-inch vials with 20 ml of food on the surface of which we had added approximately 4,000 colony-forming units (CFUs) of one of the four commensal bacterial strains (*A*. *tropicalis*, *E*. *faecalis*, *L*. *brevis*, and *L*. *plantarum*). Flies were allowed to feed on this food for one day, thereby acquiring these single-species microbial populations in their gut. To prevent the added bacteria from growing excessively on the food media, the flies were transferred to sterile food after one day where they laid eggs. For our measurements, we collected progeny from this second set of vials. These flies were never in direct contact with the initial bacterial inoculum but instead acquired their microbiome through the bacteria deposited by their parents on the eggs and food media. Before taking measurements, we allowed the flies to age for 3–5 days in a fresh, autoclaved food vial. Through all treatments, flies were maintained at 25°C with 12 hour light-dark cycles on Bloomington medium. At least two independent gnotobiotic line replicates were created for each fly-bacterium combination.

### Quantification of gut bacteria levels

We quantified bacterial load in the guts of the gnotobiotic 3–5 day old fly progeny as follows. At the flies’ “dawn”, we transferred them to fresh, autoclaved vials with sterile food media. Since flies increase their feeding rate in the morning [58], this helped ensure that flies ingested a minimal amount of external bacteria in the hours preceding their sampling. It also minimizes the amount of dead bacteria in the gut as passage through the *Drosophila* gut can occur in under 24 hours [53, 59]. The exact eating cycles and gut passage times are unknown for these individual flies, however, so it is important to note, that despite these precautions, some non-resident or dead bacteria may have remained. After 6–11 hours, flies were sexed and then washed by vortexing them for two minutes in 1.5 ml centrifuge tubes with 70% ethanol. This was followed by two 1-minute rinses in sterile water. Flies were then immediately frozen on dry ice and maintained at -80°C until DNA extraction. For each line-bacterium combination, we collected three pools of 10 flies from at least two different experimental blocks. Flies were only pooled with others from the same block.

For each biological replicate, we extracted DNA from the pooled flies using Qiagen DNeasy Blood and Tissue kits with a modified protocol. Briefly, flies were added to 96-well plates with 180 μl lysis buffer (20 mM Tris-Cl, 2 mM sodium EDTA, 1.2% Triton X-100, and 20 mg/ml fresh lysozyme), 200 μl Qiagen Buffer AL, four 2.0 mm zirconia beads, and 0.1 ml of 0.1 mm glass beads. The plates were then processed for 2 minutes on a BioSpec Mini-Beadbeater-96. Following lysis, we added 20 μl proteinase K and incubated the samples at 56°C for 3.5 hours. To ensure there would be no remaining RNA in our sample, we performed a double RNase digest with both RNase A (10 μg/ml) and RNase T1 (25 units/ml), incubating at 37°C for 30 minutes. We then added 200 μl ethanol and proceeded with the standard Qiagen spin-column protocol.

We performed quantitative real-time PCR on total genomic DNA to determine the ratio of bacterial to fly DNA in each sample. Each 10 μl reaction contained 5 μl gDNA (approximately 30 ng) and 5 μl of Roche LightCycler 480 SYBR Green I Master. Reactions were carried out on a Roche LightCycler 480 with the following protocol: 5 minutes at 95°C followed by 50 cycles of 95°C for 15 seconds, 60°C for 30 seconds, and 72°C for 10 seconds. Each qPCR plate also included negative controls (sterile water) to ensure that the assay was not contaminated. All reactions were run in triplicate. We measured the amount of *D*. *melanogaster* DNA with primers that targeted the single copy gene *Dfd* (5’-GTAGCGAAGAAACCCACCAA-3’ (For), 5’-ACGCTCCACTCACCTCATTC-3’ (Rev)). For each sample, we used a pair of bacteria-specific primers that provided greater sensitivity than universal bacteria primers. Primers used were: *A*. *tropicalis*, 5’-TAGCTAACGCGATAAGCACA-3’ (For), 5’-ACAGCCTACCCATACAAGCC-3’ (Rev); *E*. *faecalis*, 5’-TGCTTGTTGGGGTTGTAGGACTCCA-3’ (For), 5’-CGGGGCTTTCACCCTCTTTAGCG-3’ (Rev); *L*. *brevis*, 5’-TCAGTTTTGAGGGGCTTACCTCTCT-3’ (For), 5’-GGCATCCACCATGCGCCCTT-3’ (Rev); *L*. *plantarum* 5’-TGCGGCTGGATCACCTCCTTTC-3’ (For), 5’-ACTGGTTCGGTTCCAATGGGCC-3’ (Rev). Each primer pair was tested on axenic flies to ensure that it did not amplify any region of the *Drosophila* genome.

For a subset of six *A*. *tropicalis* colonized lines, we also obtained culture-dependent measurements of bacterial population size. Flies were surface sterilized then guts were rapidly dissected in sterile Ringer’s solution. Single guts were homogenized in 500 μl LB and plated on LB agar plates using a Spiral Biotech Autoplate 4000. Plates were incubated at 37°C for one day, then the number of colony forming units per gut was estimated using a QCount Colony Counter.

### Statistical analyses

For each biological replicate, we calculated the mean Ct value of the three technical replicates for each primer pair (bacteria-specific and fly *Dfd* gene). We then calculated the relative commensal bacterial level by subtracting the mean bacterial gene Ct from the mean *Dfd* Ct value. This gave us a relative measure of bacterial load that was normalized to the DNA content of the fly. Because each primer pair has a different efficiency, we did not attempt to compare absolute levels of the different bacterial strains. Rather we treated each bacterial strain as being measured with arbitrary units, and when comparing across bacteria, we based our analysis on the relative rank order of the fly lines.

To test the effect of fly genotype on bacterial level, we constructed a linear mixed model using the lme4 package in R [60]. In the model, we used fly line as a fixed effect and experimental block as a random effect. To test whether there was significant variation among fly lines for their relative level of commensal bacterial, we used R to construct an ANOVA table and perform hypothesis tests on this model. We tested for correlations between commensal bacterial levels using the function cor.test in R. To calculate the broad sense heritability (H^2^) for the trait, we constructed a random-effects linear model where block and line were random effects and relative commensal bacterial level was the response variable. We then calculated H^2^ = σ^2^_G_/(σ^2^_G_ + σ^2^_E_), where σ^2^_G_ was the variance attributed to the line effect and σ^2^_E_ was the residual variance.

### Association testing and gene set enrichment

We obtained genotype information for each of our lines from the DGRP website (DGRP Freeze 2.0; dgrp.gnets.ncsu.edu). Using PLINK [61], we filtered this variant set based on minor allele frequency (MAF > 0.1) and genotyping rate (> 0.9). After filtering, we were left with 1,455,565 SNPs and small indels. Using this filtered set, we constructed an IBS kinship matrix with EMMAX [35] to control for hidden population structure. We then used EMMAX to perform association tests. As our phenotypes, we used the mean of the residuals for each line replicate. Residuals were calculated from a linear model with line and experimental block as random effects using the R package lme4. We identified all variants that were significant at a Benjamini-Hochberg FDR of 0.05 using the p.adjust package in R and annotated the functional effect of these variants with the Ensembl Variant Effect Predictor v. 73. To carry out gene set enrichment analysis, we used all variants that associated with bacterial level at a nominal p-value below 10^−5^. Each variant was assigned to a single gene using the Ensembl annotations. In cases where the variant had multiple Ensembl annotations, we chose the one that was most likely to cause a phenotypic effect. In cases where the variant had the same predicted effect in multiple genes, all genes were carried forward into the gene set enrichment analysis. We created a background gene set by repeating this analysis for all variants that were used in the EMMAX associations. Using these test and background gene sets, we performed Gene Ontology (GO) analysis with the DAVID [41], Panther [39], and GOrilla [42] analysis programs.

### Measurement of fly gut length

Guts of 5-day-old, conventionally reared female flies were dissected in PBS and directly mounted between slide and coverslip in AF1 mounting solution (Citifluor Ltd). Guts were scanned with an LSM500 Zeiss confocal microscope acquiring the autofluorescent signal of Drosophila midguts (with broad GFP filter) and with automatic tiling to cover the entire surface of the gut. Anterior, middle, and posterior midgut lengths and widths were measured using FIJI (https://fiji.sc/) and Zen Blue (Zeiss). Three replicates of the experiment were performed for three separate fly generations.

### Measurement of ROS levels

We measured the ROS levels in the fly intestine by following the ferric-xylenol orange (FOX) assay [34]. In brief, individual guts were rapidly dissected from 3- to 5-day-old, conventionally reared male flies that had been maintained on standard glucose-yeast media. Dissections were performed in PBS with 2 mg/ml of aminotriazol. Five guts from each line were pooled in 50 μl of water containing 2 mg/ml of aminotriazol and centrifuged for 5 minutes at 3000 g. The supernatant was added to FOX reagent in the presence of 100 mM sorbitol [62]. After 30 minutes, we measured change in absorbance at 560 nm on a SpectraMax M2 running SoftMax Pro 4.8. Dissections and measurements were repeated twice in two separate blocks for a total of four replicates.

## Supporting Information

S1 Fig ROS levels vs. *L*. *plantarum* bacteria levels in the guts of male flies. The values given are the means of the model residuals after accounting for experimental variables. Each point represents a separate fly genotype.
(PDF)

S2 Fig Mean female midgut length vs. mean male midgut length in 9 fly lines. Each point represents a separate fly genotype. Male and female length measurements strongly correlate (R^2^ = 0.8781).
(PDF)

S1 Table Mean bacterial load in male flies from the 36 lines. The values given are the means of the model residuals after accounting for experimental variables.
(TXT)

S2 Table ROS levels in male fly intestines as measured with the ferric-xylenol orange (FOX) assay [34]. The values given are the means of the model residuals after accounting for experimental variables.
(TXT)

S3 Table Variants associated with bacterial load at an FDR of 0.05. (TXT)

S4 Table Significantly enriched GO terms from analysis with GOrilla and DAVID. (XLS)
