# Supplementary figures and images for: Drosophila Genotype Influences Commensal Bacterial Levels
Source: PLoS One. 2017 Jan 17;12(1):e0170332. doi: 10.1371/journal.pone.0170332 (PMC5240971; doi:10.1371/journal.pone.0170332)

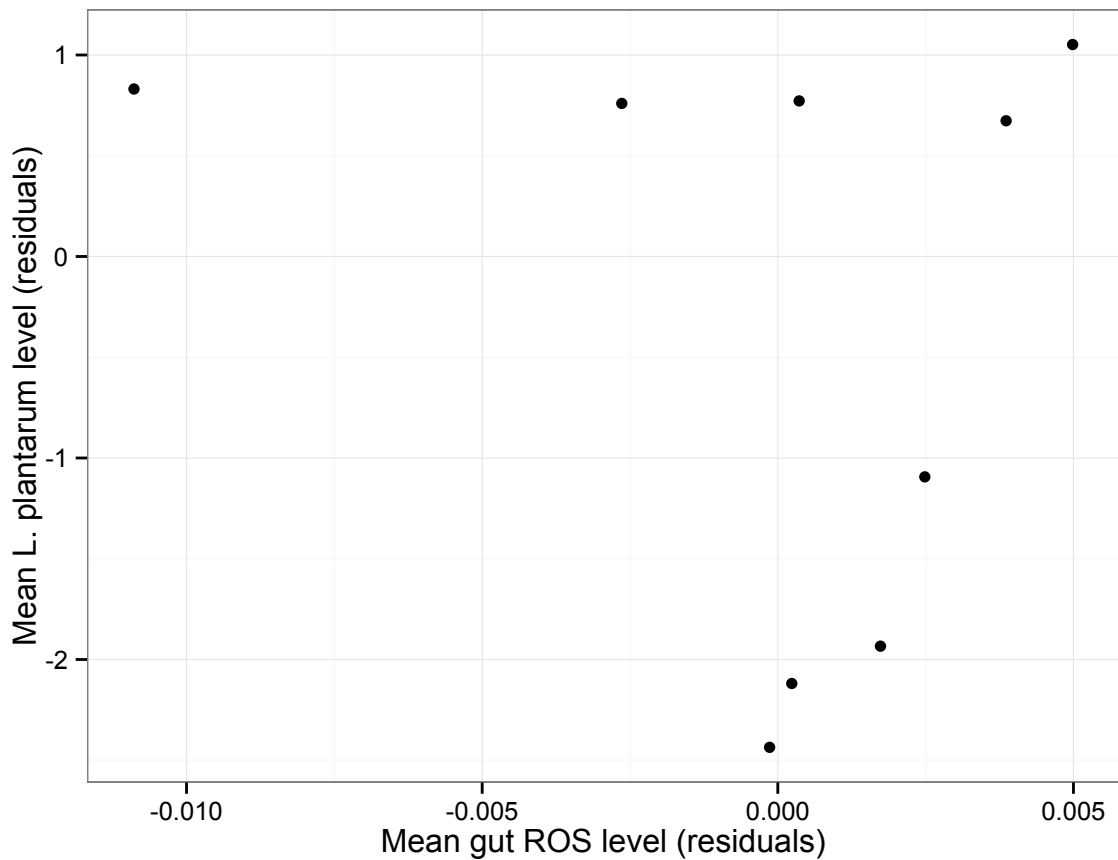

Supplement: S1 Fig — The values given are the means of the model residuals after accounting for experimental variables. Each point represents a separate fly genotype. (PDF) [file pone.0170332.s001.pdf]

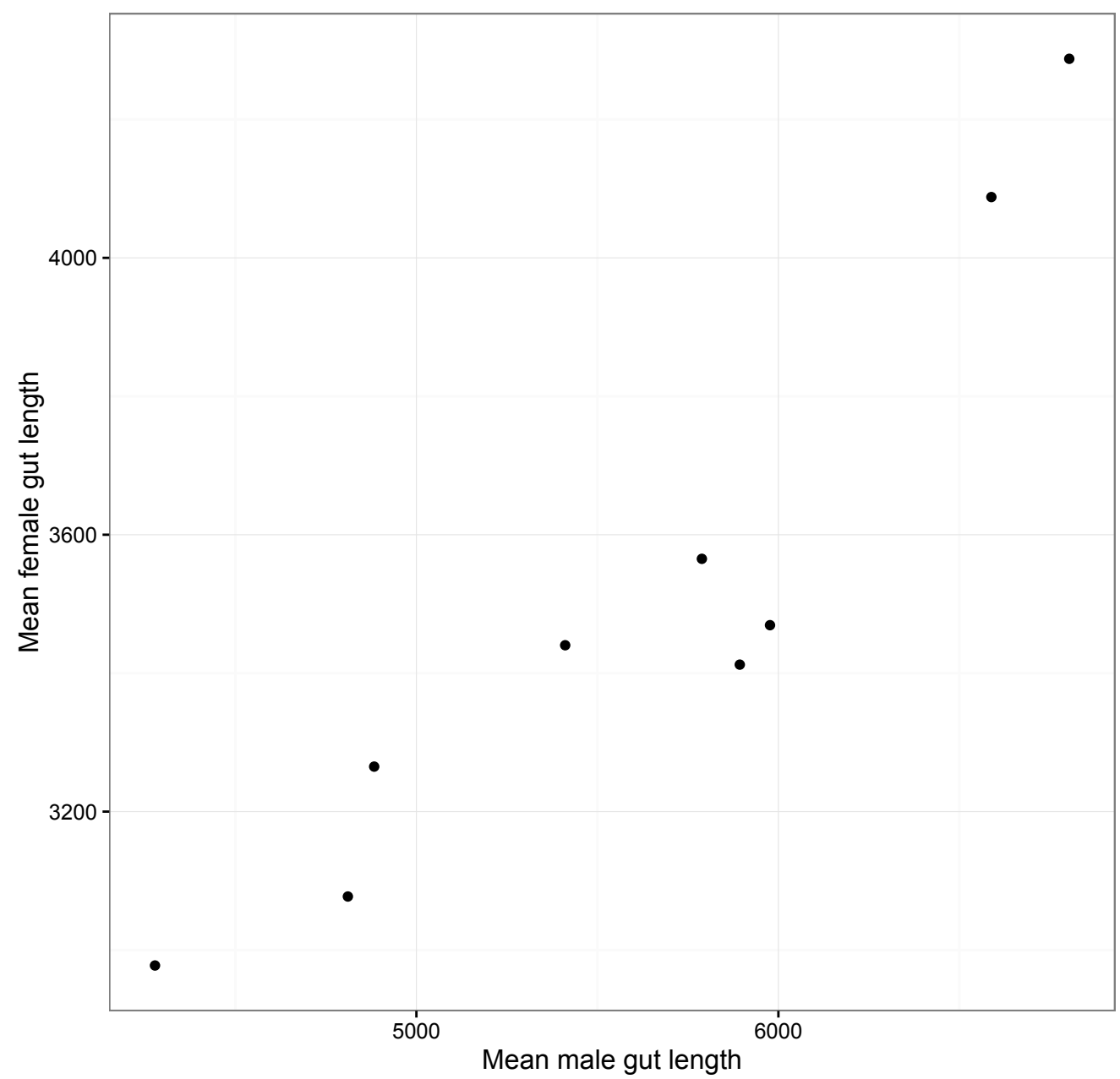

Supplement: S2 Fig — Each point represents a separate fly genotype. Male and female length measurements strongly correlate (R2 = 0.8781). (PDF) [file pone.0170332.s002.pdf]
